# Stereotactic Body Radiotherapy vs. Metastasectomy for Soft Tissue and Bone Sarcoma Lung Metastases – A Systematic Review analyzing Safety and Efficacy

**DOI:** 10.1016/j.ctro.2025.101097

**Published:** 2025-12-20

**Authors:** Lena Kretzschmar, Maksym Fritsak, Philip Heesen, Astrid Heusel, Sylvie Bonvalot, Matthias Guckenberger, Aisha Miah, Falk Röder, Maria Anna Smolle, Sebastian M. Christ, Siyer Roohani

**Affiliations:** a Department of Radiation Oncology, University Hospital Zurich and University of Zurich, Zurich, Switzerland; b Faculty of Medicine, University of Zurich, Zurich, Switzerland; c Department of Biostatistics, Epidemiology, Biostatistics and Prevention Institute, University of Zurich, Zurich, Switzerland; d Department of Surgery, Institut Curie, Paris, France; e Department of Radiotherapy and Physics, The Royal Marsden Hospital and The Institute of Cancer Research, London, United Kingdom; f Department of Radiation Therapy and Radiation Oncology, Paracelsus Medical University Hospital, Salzburg, Austria; g Institute of Research and Development of Advanced Radiation Technologies (radART), Paracelsus Medical University, Salzburg, Austria; h Department of Orthopaedics and Trauma, Medical University of Graz, Graz, Austria; i Department of Radiotherapy and Radiation Oncology, University Medical Center Hamburg-Eppendorf, Hamburg, Germany; j German Cancer Consortium (DKTK), partner site Berlin, a partnership between DKFZ and Charité - Universitätsmedizin Berlin, Germany, Heidelberg, Germany

**Keywords:** Sarcoma, Pulmonary metastasis, Oligometastatic, SBRT, Surgery, Outcome, Toxicity

## Abstract

**Background:**

Pulmonary metastases (PM) develop in ∼ 30 % of sarcoma patients after curative therapy and confer poor prognosis. Surgery and stereotactic body radiotherapy (SBRT) are viable local ablative options, but direct comparative data is limited. This systematic review evaluates oncologic outcomes and toxicities of surgery versus SBRT for sarcoma-derived PM.

**Materials and methods:**

We systematically reviewed Medline and references for studies of sarcoma-PM treated with surgery or SBRT according to PRISMA, including publications up to 13th May 2025. Outcomes included local control (LC), progression-/disease-free survival (PFS/DFS), overall survival (OS), and toxicities, with OS data harmonized for pooled analysis.

**Results:**

Fifty-eight mostly retrospective studies comprising 4,787 patients were included. LC, PFS, and DFS were heterogeneously reported but generally similar. Three-year weighted median LC was 89.6 % (surgery, 2 studies) vs. 87.8 % (SBRT, 15 studies). Reporting on PFS/DFS was limited: Four-year-PFS 21 % (SBRT) vs. 28 % (surgery, 1 study each); 5-year-DFS 12 % (SBRT, 1 study) vs. 19 % (surgery, 9 studies). SBRT toxicities were generally ≤ CTCAE grade 3, while surgery occasionally caused grade 4–5 events. 5-year-OS showed no significant difference: 31.6 % for SBRT [95 %-CI 23.9–41.5 %], 37.8 % for surgery [95 %-CI 30–41.9 %].

**Conclusion:**

Evidence indicates SBRT and surgery provide comparable outcomes for sarcoma- PM, with SBRT offering a more favorable toxicity profile. Though heterogeneous indications and incomplete reporting limit comparability, SBRT should not be viewed solely as fallback for surgically ineligible patients. Treatment should be individualized within a multidisciplinary expert team, integrating patient- and modality-specific factors. Well-designed prospective trials are needed to define relative safety and efficacy.

## Highlights

• Surgery & SBRT for sarcoma-PM achieve similar tumor control and survival metrics.
• SBRT shows a generally more favorable toxicity profile.
• SBRT should be considered a standard option independent of surgical eligibility.
• Treatment should be individualized within a multidisciplinary team of experts.
• Limitations: Heterogeneous designs, reporting, predominance of retrospective data.

## Nomenclature

**Abbreviation**
ASPS: Alveolar soft part sarcoma
BED_4_: Biologically effective dose (at alpha/beta ratio of 4)
BS: Bone sarcoma
CI: Confidence interval
CT: Computed tomography
CTCAE: Common Terminology Criteria For Adverse Events
DFS: Disease-free survival
Fig.: Figure
FU: Follow-up
ITV: Internal target volume
LC: Local control
Met.: Metastasis/-es
MPNST: Malignant peripheral nerve sheath tumor
N/A: Data not available
No.: Number
OS: Overall survival
PFS: Progression-free survival
PM: Pulmonary metastasis/-es
PRISMA: Preferred Reporting Items For Systematic Reviews And Meta-Analyses
PROSPERO: International Prospective Register Of Systematic Reviews
R0: Clear surgical margins
R1: Microscopically positive margins
R2: Gross disease
R+: Incomplete tumor resection
ROBINS-I: Risk Of Bias In Non-Randomized Studies − Of Interventions
SBRT: Stereotactic body radiotherapy
STS: Soft tissue sarcoma
UPS: Undifferentiated pleomorphic sarcoma
Yr.: Year(s)

## Introduction and background

### Rationale

Soft tissue sarcomas (STS) and bone sarcomas (BS) constitute a heterogenous group of rare mesenchymal malignancies, accounting for approximately 1 % of all adult cancers [1]. Despite effective local therapy, distant metastases—most commonly to the lungs-develop in about 30 % of sarcoma patients and are associated with poor outcomes [2], [3], [4]. For patients with pulmonary metastases (PM), local metastasis-directed treatment strategies such as surgical metastasectomy have been associated with prolonged progression-free survival (PFS) in retrospective analyses [5], [6], [7], [8]. Stereotactic body radiotherapy (SBRT), a highly precise and non-invasive local ablative treatment, was initially utilized primarily in patients with primary and secondary pulmonary malignancies who were unfit for surgery [9]. However, due to its demonstrated efficacy and favorable toxicity profile, SBRT has increasingly been considered a viable alternative to surgery for selected patients with PM [10], [11], [12]. Both treatment modalities offer distinct advantages and limitations, wherefore treatment decisions should be individualized within multidisciplinary sarcoma teams [13]. In 2020, Tetta et al. conducted a systematic review comparing these two modalities in the context of PM deriving from STS, highlighting the lack of randomized phase III trials as a major knowledge gap in the field [14]. Since then, the evidence base has expanded, particularly for STS and BS, yet no direct prospective comparisons exist. In light of this, a comprehensive understanding of the available data for each treatment modality is essential to guide clinical decision-making.

### Objectives

The objective of this systematic review was to investigate key outcomes of surgical and SBRT treatments of PM deriving from sarcoma, including local control rate (LC), disease--free survival (DFS), PFS, overall survival (OS) and treatment-related toxicities. It aims to give a structured overview of the available body of evidence and seeks to compare the two treatment modalities to guide treatment recommendations.

## Materials and methods

### Protocol and registration

This systematic review was conducted following the current *Preferred Reporting Items for Systematic Reviews and Meta-Analyses (PRISMA)* guidelines [15]. The review was prospectively registered in the *International Prospective Register of Systematic Reviews (PROSPERO)* on 29th April 2025 [16]. A full version of the published review protocol (“Systematic Review on the Precision Treatment of Pulmonary Sarcoma Metastases – Stereotactic Body Radiotherapy vs Metastasectomy”, Version 1.3 / ID CRD420251037919) is available in the Supplementary Files. As this research was based solely on publicly available data, institutional review board approval was not required.

### Eligibility criteria

All original articles in English language published before 13th May 2025 reporting on patients with one or more PM from any type of sarcoma receiving locally ablative treatment with surgery, SBRT, or both, were included. The following types of studies were included: randomized and non-randomized controlled trials, retrospective and prospective observational studies involving SBRT or metastasectomy for sarcoma-derived PM. Review articles, guidelines, letters to the editor, opinions or editorials, additional publications on an existing patient cohort, studies focusing on other locally ablative techniques such as radiofrequency ablation, and studies that contained sarcoma patients with PM but focused on different aspects (such as treatment of the primary tumor), thus failing to report on outcome measures of interest to this review, were excluded. Studies including PM of other histologies than sarcoma could be included, if relevant outcome measures for sarcoma patients were explicitly reported as a subgroup analysis (treatment efficacy outcomes or toxicities or both). While case report series were integrated in the review, single-patient case reports were excluded as well. Stratification of sarcoma entities was performed according to the 2020 WHO Classification of Tumours of Soft Tissue and Bone [1]. Although STS and BS are biologically and clinically distinct entities with differing metastatic patterns and treatment sensitivities, the rarity of sarcoma and the limited, heterogeneous evidence base necessitate a broader approach [1], [4]. Most available studies also combined BS and STS and often lacked subtype-specific reporting, making separate analyses infeasible. We therefore synthesized outcomes across all sarcoma subgroups to provide the most comprehensive assessment possible.

### Information sources and search strategy

Data were primarily extracted from *Medline* using the search term “*(“radiotherapy”[MeSH Terms] OR (stereotactic AND radiotherapy) OR (“metastasectomy”[MeSH Terms]) AND (“lung neoplasms/secondary”[MeSH Terms]) AND (“sarcoma”[MeSH Terms])*”; this query yielded 319 abstracts that were then semiautomatically preprocessed. During preprocessing, a custom Python script parsed the abstracts and retained only those that included “sarcoma” to define the disease, “lung” or “pulmonary” to define the location, and “sbrt,” “stereotactic,” “surgery,” or “metastasectomy” to define the intervention. This process was followed by a brief manual review to remove false positives and double-check against false negatives, resulting in a set of 81 studies. In a second step, an additional manual reference search was performed on these 81 studies, utilizing *Google Scholar* to branch out into other large electronic databases such as *Cochrane Central* and *Scopus*. With this search strategy an additional 20 studies relevant to the review were identified, adding up to a total number of 101 studies for screening. Full, electronic text files could be retrieved for all these studies.

### Selection process

**Fig. 1:**
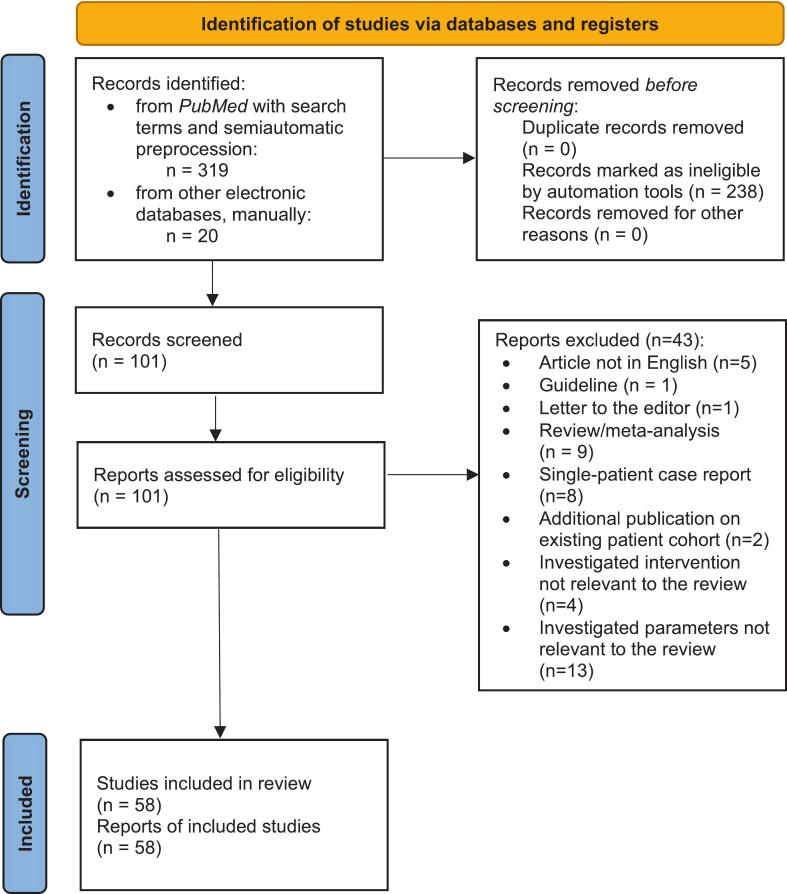
PRISMA Flow-Diagram of study selection process.

After retrieval of full text files for all studies, two independent researchers (L.K., A.H.) conducted a thorough eligibility assessment based on the inclusion criteria described above by systematically reading and annotating the full articles. Of 101 studies, a total of 58 studies were included in the final analysis (a detailed list of excluded studies and reasons for exclusion is outlined in Fig. 1).

### Data collection process and data items

Data collection was conducted manually by the same two reviewers using a standardized extraction form developed to ensure consistency across records. The reviewers worked independently, with each responsible for a prespecified subset of studies. The following parameters were extracted for all included studies: Title, publication year, name of first author, study design, number of patients (total/treated with surgery (if any)/treated with SBRT (if any)), number of PM (total/treated with surgery (if any)/treated with SBRT (if any)), subtype of sarcoma, inclusion of other histologies besides sarcoma, size of PM, *peri*-interventional systemic therapy, median follow-up, LC rate, PFS, DFS, extrapulmonary systemic failure-free survival, OS, and toxicity according to the Common Terminology Criteria for Adverse Events (CTCAE) and the Clavien-Dindo classification [17], [18]. Tumor control/survival data was collected at specific time points (e.g. 1-year, 3-year, etc.); all time points explicitly stated in the respective studies were recorded. In addition, for studies containing patients treated with surgery, the type and extent of surgery, as well as the surgical completeness of pulmonary clearance was collected for the individual metastases and per patient. For studies containing patients treated with SBRT, radiotherapy details such as prescription (including physical dose and biologically effective dose at alpha/beta ratio of 4 (BED_4_), number of fractions, isodose level) and SBRT technique, were recorded. Missing information was clearly marked as such in the extraction form. After completion of data collection, a senior investigator independently screened the data sheet for consistency and completeness.

### Study risk of bias assessment

Due to the absence of randomized studies in the analysis, risk of bias was assessed exclusively using the Cochrane *ROBINS-I* tool and visualized with the *robvis* tool [19], [20]. Assessment was conducted independently by two investigators with availability of a third (senior) investigator to resolve any differences in assessment of outcomes.

### Effect measures and synthesis methods

We primarily used descriptive statistical analysis to compare studies. This included frequencies and percentages for categorical and ordinal variables such as missingness of data (item reported vs. not reported), study design (retrospective vs. registry-based vs. prospective observational vs. prospective interventional), systemic therapy, surgical completeness of pulmonary clearance (only fully resected metastases vs. non-fully resected metastases present in the cohort), type of SBRT treatment, toxicity data and sarcoma histology. The latter was divided into three categories: STS, BS, and mixed/undefined sarcoma entities – each study was sorted into one category depending on its patient cohort. For continuous variables (e.g. number of treated patients per study, number and size of treated metastases), medians and (interquartile) ranges were used.

The data points LC, PFS, DFS and extrapulmonary failure-free survival were reported infrequently and at various time points across studies and thus only lent themselves to a descriptive analysis. A more intricate statistical comparison between surgery and SBRT groups was only conducted for OS data, which was reported more frequently. To compare OS between surgery and SBRT groups, we assumed a constant hazard rate across studies:

$$S\left(t\right)=e^{-\lambda t}$$

so that OS proportions (S) reported at different follow-up times (t) could be harmonized by estimating a single hazard rate (λ) per study. Taking into account the different number of patients involved in each study to avoid publication bias (small study bias), we calculated a weighted mean λ, where the weights correspond to the number of patients in a given study divided by the total number of patients in the respective treatment group (surgery or SBRT). To estimate 95 % confidence intervals, we applied bootstrapping with 10,000 repetitions, using selection probabilities equal to the previously derived study weights. We then compared OS curves between surgery and SBRT using a Wald test based on the logarithmic rate ratio, with statistical significance defined as *p* ≤ 0.05. A two-sided Mann-Whitney-U-Test was used to assess the stochastic differences between the follow-up times for surgery and SBRT. Statistical analyses were conducted using Python (version 3.12.4) with the NumPy (version 1.26.4) and Pandas (version 2.2.3) packages, as well as R (v.4.4.2, R Core Team 2024) and R Studio (v2025.05.0, Posit Software 2025), led by P.H. and M.F.

### Protocol Deviations and Clarifications

While the initial PROSPERO Protocol (ID: CRD420251037919) was published on 29th April 2025 (Version 1.0), a total of three minor revision have been made to the protocol. Version 1.1 was published on 20th May 2025, Version 1.2 on 21st July 2025, and the last and final Version 1.3 on 15th August 2025. All revisions were confined to the “Current Review Stage”, updating the progress made on the Systematic Review. One major deviation from the protocol needs to be addressed as follows: While the protocol explicitly does not exclude non-English-language articles, studies included in our systematic review were eventually limited to English-language-only publications. This decision was made in the late stage of full-text extraction due to uncertainties in extracted information from non-English language publications with available software, and did not enter into the protocol.

## Results

### Study selection

A total of 58 publications were analyzed, primarily retrospective studies (n = 51) [[5], [11], [21], [22], [23], [24], [25], [26], [27], [28], [29], [30], [31], [32], [33], [34], [35], [36], [37], [38], [39], [40], [41], [42], [43], [44], [45], [46], [47], [48], [49], [50], [51], [52], [53], [54], [55], [56], [57], [58], [59], [60], [61], [62], [63], [64], [65], [66], [67], [68], [69]]; followed by registry-based (n = 3) [[70], [71], [72]]; prospective interventional, non-randomized (n = 2) [[12], [73]]; and prospective observational studies (n = 2) [[74], [75]]. The selection process for the studies is shown in the Flow-Diagram above (Fig. 1). An overview of all studies including n ≥ 20 patients is displayed in Supplementary Table 1.

### Study characteristics and number of patients

**Fig. 2:**
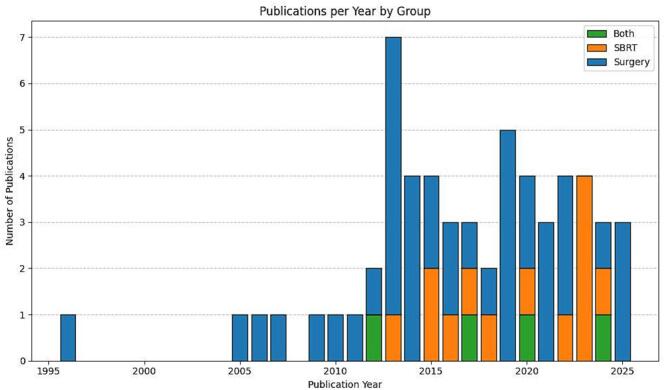
Distribution of publications by year of publication, stratified by surgery- and SBRT-based reports.

**Fig. 3:**
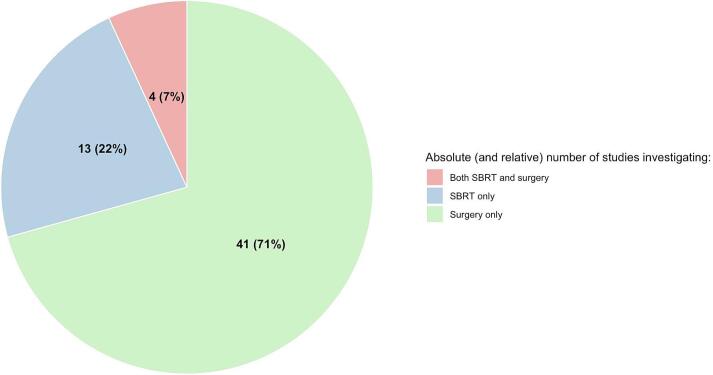

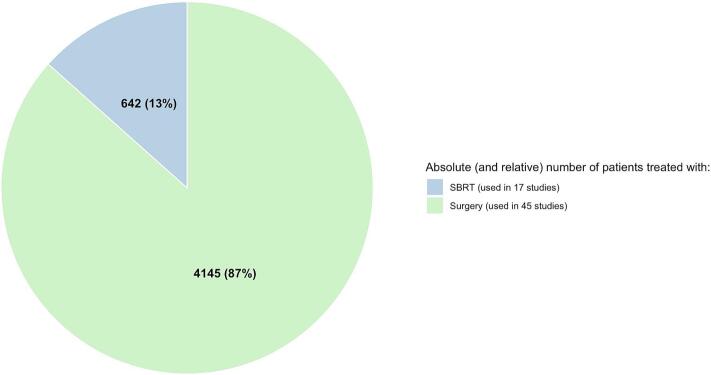

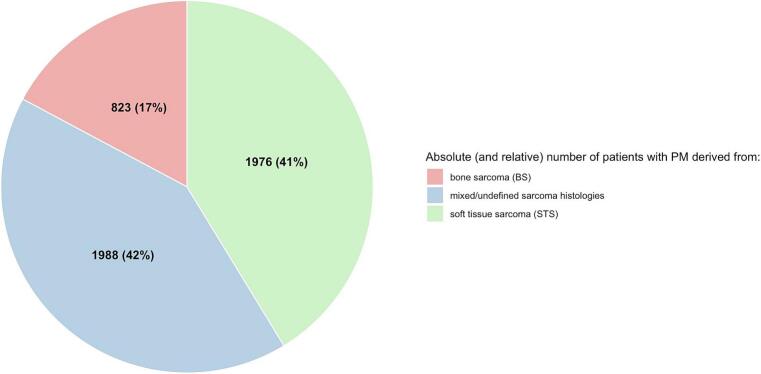
Proportion of publications by investigated treatment modality (A); Proportion of patients by treatment modality across publications (B); Proportion of patients with metastases arising from bone versus soft tissue tumors (C).

The publication years of included articles mainly ranged from 2005 to 2025 (Fig. 2). Across all studies, the median cohort size was 61 patients (range 7–539). Four publications reported on both surgery and SBRT [[29], [40], [50], [64]]; while 41 focused on surgery only [[5], [21], [22], [23], [25], [26], [27], [28], [33], [34], [35], [36], [37], [38], [39], [42], [43], [44], [45], [46], [47], [49], [52], [54], [56], [57], [58], [59], [60], [61], [62], [63], [65], [66], [67], [68], [69], [70], [71], [72], [73]]; and 13 on SBRT only [[11], [12], [24], [30], [31], [32], [41], [48], [51], [53], [55], [74], [75]] (Fig. 3A). Surgery-based studies had a median cohort size of 61 surgically treated patients, whereas SBRT-based studies had a median of 33 patients treated with SBRT. For distribution of patients across the treatment modalities see also Fig. 3B.

Thirty-eight percent of all studies reported the total number of treated PM [[11], [12], [23], [24], [25], [30], [31], [32], [33], [34], [38], [41], [48], [51], [52], [53], [55], [57], [58], [65], [74], [75]] (surgery-based: 9 reports [[23], [25], [33], [34], [38], [52], [57], [58], [65]]; SBRT-based: 15 reports [[11], [12], [24], [29], [30], [31], [32], [41], [48], [51], [53], [55], [64], [74], [75]]). In the studies reporting this parameter, the mean number of PM per patient ranged from 2 to 10 (median: 4) in surgery-based studies, and 1–5 (median: 2) in SBRT-based studies.

### Histologies and size of metastases

Most studies included mixed sarcoma types (n = 28) [[5], [11], [12], [24], [26], [28], [29], [30], [31], [32], [41], [43], [46], [47], [48], [49], [51], [53], [55], [56], [57], [58], [59], [63], [69], [71], [73], [74]]. Twenty studies reported STS only [[21], [22], [27], [35], [36], [38], [39], [44], [52], [54], [60], [61], [64], [65], [66], [67], [68], [70], [72], [75]]; 10 studies reported BS only [[23], [25], [33], [34], [37], [40], [42], [45], [50], [62]] (Fig. 3C). Within the entire collective of 58 studies, three studies included sarcomas alongside other histologies (e.g., endometrial, papillary serous, colorectal carcinomas) with separate outcome analyses for PM derived from sarcoma [[21], [49], [73]]. Metastasis size was highly variable throughout the collective and reported either in cm (all surgery-based studies) or cc (most SBRT-based studies): for surgical cases, metastases ranged from 0.1 to 19 cm; for SBRT, lesion sizes ranged from 0.1 cc to 375.8 cc. Data on metastasis size were incomplete in 68.9 % of surgery-based studies [[5], [21], [22], [28], [29], [33], [36], [37], [38], [39], [40], [42], [43], [44], [45], [49], [54], [56], [57], [58], [59], [63], [64], [65], [66], [67], [68], [69], [70], [72], [73]]; and 23.5 % of SBRT-based studies [[31], [40], [51], [64]].

### Treatment characteristics

Surgery:

Surgical approaches varied across studies. Most commonly, patients were not confined to one type of surgery, but instead received thoracoscopy, thoracotomy, sternotomy or a combined approach as chosen by the treating surgeon (variable surgical approaches reported in 34 studies [[5], [21], [22], [23], [25], [26], [27], [28], [34], [35], [36], [37], [38], [39], [42], [43], [44], [46], [47], [49], [52], [54], [56], [57], [59], [60], [62], [63], [67], [68], [69], [70], [71], [72]]). Seven studies reported on a thoracotomy-only approach [[33], [45], [50], [58], [61], [65], [73]]; while in 4 studies, information on surgical approach was missing [[29], [40], [64], [66]]. Regarding surgical completeness of pulmonary clearance, most studies included patients with positive resection margins (R+, 28 studies [[22], [25], [28], [34], [35], [36], [37], [38], [42], [44], [46], [47], [52], [54], [57], [58], [59], [60], [61], [63], [65], [66], [67], [68], [69], [70], [71], [72]]), indicating that complete tumor clearance was not achieved in all cases. Only 4 studies explicitly only included patients with clear margins (R0) [[23], [26], [27], [56]]. In 13 studies, completeness of excision was not reported [[5], [21], [29], [33], [39], [40], [43], [45], [49], [50], [62], [64], [73]].

Stereotactic body radiotherapy:

SBRT was delivered using a diverse range of total prescribed physical doses from 20 to 60 Gy, with most studies reporting prescribed doses in the range of 30 to 60 Gy. Fractionation schedules ranged between 1 and 12 fractions, reflecting variability in treatment protocols. As a consequence of these wide-ranging dose prescription and fractionation schemes, BED_4_ also proved diverse, spanning values from 60 − 360 Gy (see Supplementary Table 1). The most commonly reported SBRT-technique was the internal target volume (ITV) approach, used in 13 studies [[11], [12], [24], [29], [30], [31], [32], [41], [48], [51], [53], [74], [75]]. More rarely, a 4D-tumor-tracking approach was utilized, or patients were treated in breath hold. Only one SBRT-based study did not report treatment technique [40].

Systemic therapy:

The use of systemic therapy in combination with local treatments for PM was inconsistently reported for both surgery and SBRT. Only three studies provided detailed information about the number of patients receiving systemic therapy and the treatment context (neoadjuvant, concomitant, or adjuvant), including the type of agents used [[33], [45], [61]]. The majority of studies mentioned only that chemotherapy had been administered to some patients. Use of immunotherapy or targeted therapies was mentioned in only one, surgery-based, study [25].

### Toxicities

Surgery:

Surgery-related toxicities were reported in grades according to the CTCAE and/or Clavien-Dindo classification in 17 studies that contained surgically treated patients (38 %) [[23], [25], [26], [27], [35], [37], [44], [50], [52], [54], [56], [57], [58], [66], [68], [69], [73]]: Nine of the studies reported grade 5 toxicity (CTCAE/Clavien-Dindo) as the highest occurring complication level (with a total of 9 surgery-related deaths overall) [[37], [44], [52], [54], [56], [58], [66], [68], [69]]. In the context of CTCAE, 3 studies reported grade 4 toxicities as highest complication [[26], [50], [73]]; 2 studies reported grade 3 [[27], [57]]; 2 studies grade 2 [[23], [35]]. In the context of Clavien-Dindo, 4 studies reported grade 4 toxicities as highest complication [[25], [26], [50], [73]]; 3 grade 3 [[23], [27], [57]]; one grade 2 [35]. Common toxicities were prolonged air leakage, pneumonia, pneumothorax, wound infections and cardiac toxicities such as arrhythmia.

Stereotactic body radiotherapy:

SBRT-related toxicity was reported in grades according to CTCAE in 16 studies that included patients treated with RT (94 %) [[11], [12], [24], [29], [30], [31], [32], [41], [48], [50], [51], [53], [55], [64], [74], [75]]: Four studies reported grade 3 toxicity as the highest occurring complication level [[31], [32], [48], [51]]; 10 reported grade 2 [[11], [12], [29], [30], [41], [50], [53], [55], [64], [75]]; 2 reported grade 1 [[24], [74]]. No grade 4 or 5 toxicities were observed. Common toxicities were radiation pneumonitis, chest wall pain and, more rarely, esophagitis.

### Follow-up and local control

Median follow-up across the 49 studies reporting this parameter was 33 months (surgery: 33 months; SBRT: 34 months), with no significant difference between modalities (p = 0.61). Definitions of LC varied substantially among surgical series. In some, progression in either the ipsilateral or contralateral lung was categorized as local failure or simply summarized as “pulmonary failure/recurrence” [[37], [38], [42], [43], [47], [52]]. Only two studies reported local disease control specifically at the level of the treated region (“regional”), representing the closest anatomic definition to the treated metastasis and, thus, serving as the most accurate stand-in for the LC endpoint for the surgery-based studies included in this dataset. In contrast, 15 SBRT studies reported LC specifically at the level of the treated metastasis (“per-met”). LC was high for both approaches: Surgery showed 89–90 % at 3 years (reported on a regional level in 2 studies [[29], [73]]), whereas SBRT demonstrated median LC of 95.1 % (range: 86.0 %−100.0 %) at 1 year and 87.8 % (range: 78.1 %−98.5 %) at 3 years (reported on a per-met level in 15 studies [[11], [12], [24], [29], [30], [31], [32], [41], [48], [51], [53], [55], [64], [74], [75]]). Extrapulmonary systemic failure-free survival was reported in only one study investigating both surgery and SBRT, with nearly identical 3-year outcomes for both modalities (surgery: 61.6 %, SBRT: 60.1 %) [29].

**Progression- and disease-free survival**

In two surgery-based studies with available data, PFS was 25.0 % at 5 years (n = 1 [45]) and 27.5 % at 4 years (n = 1 [50]). In two SBRT-based studies with available data, PFS was 52.7 % at 1 year, 20.6 % at 2 years, and 12.9 % at 3 years (n = 1 [32]); and 21.2 % at 4 years (n = 1 [49]). For surgery, DFS ranged widely across studies, with 36.0 % at 1 year (n = 1 [63]), 56.7 % at 2 years (n = 1 [61]), 17.0–42.0 % at 3 years (n = 3 [[5], [69], [73]]), 19.0 % at 4 years (n = 1 [27]), and 5.0–38.0 % at 5 years (n = 9 [[5], [33], [37], [43], [46], [56], [66], [69], [71]]). For SBRT, one study reported DFS of 50.0 % at 1 year, 19.5 % at 2 years, and 11.7 % at 3–5 years [12]. Despite these individual reports, the majority of studies did not provide time-specific PFS- and DFS-data. PFS was missing for surgery in 43 studies, for SBRT in 15 studies. DFS was unreported for surgery in 32 studies and for SBRT in 16 studies.

### Results of overall survival synthesis

**Fig. 4:**
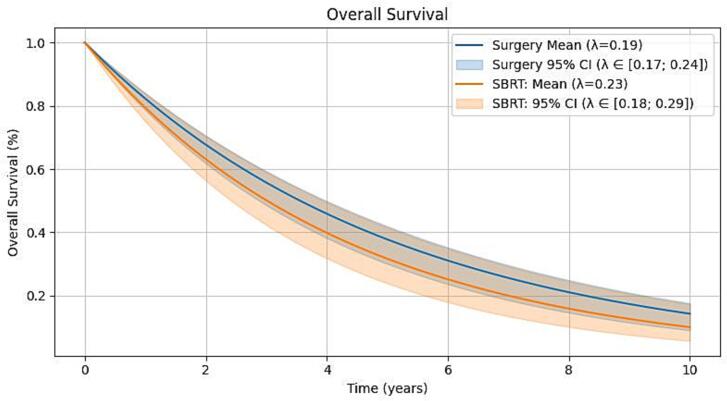
Overall survival synthesized across studies, stratified by surgery versus SBRT.

OS (Fig. 4) was reported in almost all studies (only four for surgery and two for combined SBRT/surgery studies were missing [[25], [26], [38], [50], [58], [64]]), with a weighted mean λ of 0.19 for surgery (95 % CI 0.17–0.24) and 0.23 for SBRT (95 % CI 0.18–0.29).

The two studies judged to be at critical risk of bias according to the ROBINS-I-assessment detailed further below were excluded from this pooled analysis [[51], [62]]. A more descriptive, graphical overview of OS over all studies in the dataset (including those at critical risk of bias) can be found in the Supplementary Files.

The Wald test yielded p = 0.27, indicating no statistically significant difference in OS between surgery and SBRT. The 1-year OS was 82.3 % for surgery (95 % CI 78.6 – 84 %) and 79.4 % for SBRT (95 % CI 75.1 – 83.9 %); 2-year OS was 67.7 % for surgery (95 % CI 61.8 – 70.6 %) and 63.1 % for SBRT (95 % CI 56.4 – 70.4 %) and 5-year OS was 37.8 % for surgery (95 % CI 30 – 41.9 %) and 31.6 % for SBRT (95 % CI 23.9 – 41.5 %).

Given the biological and clinical differences between STS and BS we conducted a small, exploratory survival sub-analysis on the subset of studies that investigated either only patients with STS or only patients with BS to gain a better insight into the potential for confounding that arises from these differences. For the sake of consistency the two studies judged to be at critical risk of bias were once again excluded from the analysis, leaving 20 studies that reported on STS only [[21], [22], [27], [35], [36], [38], [39], [44], [52], [54], [60], [61], [64], [65], [66], [67], [68], [70], [72], [75]], and 9 studies that reported on BS only to be analyzed [[23], [25], [33], [34], [37], [40], [42], [45], [50]]. When comparing the 1-, 2-, and 5-year OS between the two groups, no relevant differences were observed: Studies including patients with only STS reported 1-year OS of 81.0 % (95 % CI 78.3– 83.5 %), 2-year OS of 65.6 % (95 % CI 61.4– 69.7 %) and 5-year OS of 34.8 % (95 % CI 29.5– 40.5 %); while studies including patients with only BS reported 1-year OS of 80.9 % (95 % CI 65.4– 85.6 %), 2-year OS of 65.4 % (95 % CI 42.8– 73.3 %) and 5-year OS of 34.6 % (95 % CI 11.9– 46.0 %). p-value was 0.98, indicating no statistical significance. A graphical depiction of this sub-analysis can be found in the Supplementary Files.

### Risk of bias assessment

**Fig. 5:**
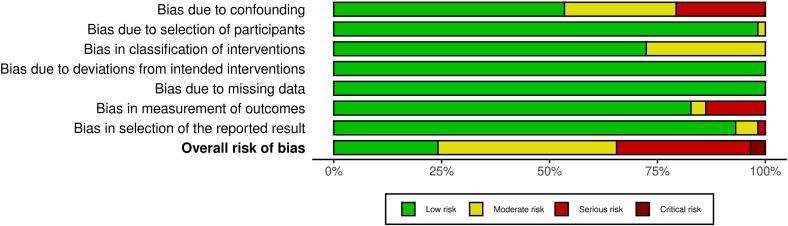
ROBINS-I risk of bias summary.

As depicted in Fig. 5, most of the 58 studies included were assessed as having low or moderate overall risk of bias (n = 38) [[5], [11], [12], [21], [25], [26], [27], [28], [29], [30], [31], [32], [33], [35], [36], [37], [39], [41], [43], [45], [46], [47], [48], [50], [53], [55], [56], [57], [59], [61], [65], [67], [69], [70], [72], [73], [74], [75]]. However, a total of 20 studies showed serious (n = 18) or critical (n = 2) risk scores [[22], [23], [24], [34], [38], [40], [42], [44], [49], [51], [52], [54], [58], [60], [62], [63], [64], [66], [68], [71]]. The two studies judged to be at critical risk were not severely biased in any of the domains, but at serious risk in several domains, leading to a higher overall risk score in the final judgement [[51], [62]]. Most “serious risk” judgements were made in domain 1 (“bias due to confounding”) due to incomplete or unclear consideration or control of confounding factors. The domain with the second highest “serious risk” count was domain 6 (“bias in measurement of outcomes”), owing to the fact that several studies retrospectively covered a long time period to compile a higher number of patient records (e.g. 40 years, from 1971 to 2011). In these timespans the standard of care diagnostic methods for detection of disease progression changed (e.g. from chest x-ray to chest computed tomography), leading to a relevant detection bias from different sensitivities of the methods. A comprehensive, study-by-study overview of the ROBINS-I-assessment can be found in the Supplementary Files.

## Discussion

Herein, we systematically reviewed 58 studies including 4,787 patients with PM deriving from sarcoma (4,145 surgery; 642 SBRT). Overall, both metastasectomy and SBRT achieved high and comparable LC rates (roughly 90 % at 3 years). Toxicities appeared more frequent and severe after surgery, though reporting—especially in surgical studies—was limited. Mortality was comparably high in both cohorts, largely driven by the frequent and comparable rates of disease progression outside the treated lesions in the lungs across both modalities.

SBRT achieved similar high control rates compared to surgery based on the available data. Reporting on LC was generally limited, although more consistent in SBRT studies, while surgical series often lacked standardized documentation. As briefly noted in the Results section, LC in a surgical context is not directly comparable to LC in a radiotherapy context: When a lesion is resectable, surgery removes the entire tumor, making the concepts fundamentally different from each other. Therefore, our closest approximation to LC in surgery-based studies was given by publications reporting “regional control” of PM, which served as a stand-in for LC in our analysis. This limits direct comparability yet does not detract from the favorable LC outcomes observed with SBRT. Notably, the only prospective phase II trial evidence from Navarria et al. achieved an excellent primary endpoint of LC (98.5 % 1-year LC) with no grade 3 or higher toxicity [12]. As expected in a metastatic malignancy, PFS and DFS were generally low, and both modalities performed similarly. This finding is not surprising, since both are local therapy modalities and systemic progression is the dominant driver of outcome. Moreover, comparisons were limited by heterogeneous reporting, wide ranges of values given across timepoints, and confounding influences from sarcoma subtypes and systemic therapy use.

OS was the most frequently reported endpoint, often with multiple time points per study. After adjustment for small-study bias and exclusion of studies at critical risk of bias in the pooled analysis [[51], [62]], surgery showed a slight numerical advantage over SBRT, though this was not statistically significant. The previous systematic review by Tetta et al. raised differences in follow-up duration as a potential confounder, but our analysis did not confirm any significant follow-up duration imbalances between the groups [14]. Despite the multitude of confounding factors affecting OS, surgery and SBRT appear comparable in this respect. Further confounding influences may be found in the diversity of the sarcoma entities included in the review dataset: When stratifying the total number of 29 studies from the pooled analysis that reported only on one subtype of sarcoma (STS [[21], [22], [27], [35], [36], [38], [39], [44], [52], [54], [60], [61], [64], [65], [66], [67], [68], [70], [72], [75]] or BS [[23], [25], [33], [34], [37], [40], [42], [45], [50]]), however, irrespective of treatment type, no relevant differences were found in OS between the two groups. This observation – at first seemingly in contradiction to the diverse population and clinical trajectory these sarcoma subtypes are typically associated with – can be explained by the diversity in study design and disease stage across the individual studies, leading to in-group heterogeneities that constitute confounding factors for the analysis.

Toxicity reporting was heterogeneous across studies. Data on SBRT-related adverse events were generally more consistently and systematically captured, whereas surgical series provided less detailed accounts. Even so, the available reports suggest that SBRT toxicities were typically limited to grade 3 or lower, while surgery was associated with higher-grade complications, including grade 4 and 5 events. The most severe surgical toxicities were often linked to high-risk procedures or surgical revisions. These findings need to be interpreted carefully, as the limited number of reports in the surgical series raises the possibility of reporting/sampling bias. In addition, a more in-depth morbidity analysis with stratification by invasiveness of utilized surgical procedure (thoracoscopy vs. thoracotomy, wedge resection vs. lobectomy) was not feasible in the scope of this review due to the frequent mixing of different surgical approaches within study cohorts and lack of procedure-specific reporting of surgery-related morbidity. Nevertheless, the observation that toxicity rates appeared higher for surgery despite fewer available reports is interesting and should be taken into consideration when making treatment decisions. Additionally, SBRT cohorts often included medically inoperable patients with comorbidities, yet their survival outcomes remained largely comparable to those of surgically treated patients, as mentioned above – raising the possibility that a treatment with more favorable toxicity profile may help balance baseline risk in this group.

In this context, four included studies that directly compared surgical and SBRT-based approaches and thus represent the closest approximation to a direct comparison between the two modalities within our dataset may be highlighted [[29], [40], [50], [64]]. Though a direct comparison between these studies is not feasible due to differences in design, it should be noted that three of them concluded comparability in the results for their respective endpoints (LC, PFS, OS) between both modalities [[29], [50], [64]]. The fourth study (focusing exclusively on patients with chondrosarcoma) observed significantly longer OS for patients receiving metastasectomy than those receiving SBRT – however, this study explicitly allocated only inoperable patients with low performance scores to the SBRT group, which could serve to explain this observation [40].

### Implications

Our findings have several implications for clinical practice and future research. The important issue of patient selection must be addressed. Both surgery and SBRT provide excellent LC for PM, but many patients eventually experience systemic progression and die from their disease. The critical question remains: which patients with sarcoma truly benefit from local ablation of PM? Though efforts to define oligometastatic disease have previously been undertaken in a more general, non-sarcoma-specific setting, sharpening and fitting criteria of oligometastatic disease for sarcoma patients or even sarcoma-subtype-specific patients would be an important step towards answering this question [76]. While this review—spanning diverse sarcoma subtypes and heterogeneous oligometastatic states—did not stratify outcomes by oligoprogression, number of treated lesions, or extrapulmonary disease, these factors may influence prognosis. Developing a unified, expert-endorsed definition of oligometastasis in sarcomas is essential, and future trials should adopt standardized criteria to clarify how metastatic burden and distribution affect SBRT and metastasectomy outcomes.

While improving OS remains the most meaningful, overarching goal, smaller goals such as longer systemic-therapy–free survival, i.e. keeping patients off toxic systemic treatments for longer periods by means of effective local therapy, may already represent a significant clinical benefit.

Moreover, our results indicate that SBRT should not be regarded merely as a fallback option for patients considered unfit for surgery. Current practice reserves SBRT for those at high risk of surgery-related morbidity. This includes patients in whom the surgical procedure itself is more fraught with risk, such as those planned for complex bilateral resections, or resections of PM adjacent to critical mediastinal structures including the heart or proximal bronchial tree. SBRT is also considered for patients with recurrent PM who have undergone multiple prior lung surgeries or might be at risk for tumor re-recurrence due to incomplete (R1/R2) resections, resulting in compounding of perioperative risks. Additionally, it may be preferred in patients for whom postoperative complications or prolonged recovery times are anticipated, such as older adults or those with significant chronic comorbidities.

In all of these cases, using SBRT instead of surgery is a reasonable approach. Beyond these indications, available data suggest that SBRT is at least as effective as, and potentially less toxic, than surgery, challenging its reputation as a “second-choice” modality, which it has been perceived as in the past [77]. Furthermore, in light of the favorable toxicity spectrum observed with SBRT, future efficacy may be enhanced by dose escalation. Notably, many sarcoma PM in our analysis received relatively low doses (BED_4_ < 175 Gy), whereas contemporary literature indicates that doses BED_4_ ≥ 175 Gy are associated with improved local control[78]. The choice of one technique or another depends on the patient’s characteristics (performance status, comorbidities, etc.), the number, size, and location of the metastases, and whether it involves a repeated local treatment of metastases or not, owing to the specific advantages and disadvantages of each technique. This observation aligns with findings from oligometastatic settings of other primary tumors, such as non-small-cell lung cancer and prostate cancer, where SBRT to the oligometastatic sites has consistently shown excellent results with regards to longer systemic-therapy-free survival, or even OS improvement [79], [80], [81], [82]. However, SBRT as a treatment option for sarcoma-derived PM has several limitations. Challenges for irradiation often present themselves in patients requiring histological samples of PM to guide systemic therapy, in those with very large lesions (>6 cm) and in patients with inflammatory or interstitial lung disease, though this patient group also poses challenges in terms of operability, often leaving SBRT as the only viable treatment option [83]. Individualizing treatment decisions by weighing the advantages and disadvantages of both surgery and SBRT against each other is, therefore, of paramount importance. Even treating different metastases in the same patient sequentially by surgery and SBRT can be a valuable option depending on the situation [13]. Therefore, the multidisciplinary context at expert sarcoma centers remains central to management. Treatment decisions for patients with PM should integrate input from surgery, radiation oncology, and medical oncology, and ideally include structured, joint consultations with patients to support informed decision-making that balances risks, benefits, and patient preferences.

Finally, given the rarity and heterogeneity of sarcoma, systematic, multi-institutional efforts are required to strengthen the evidence base for management of oligometastatic disease; a fact that has been commented on in several reviews of existing data in the past [13], [77]. Particularly the variability in radioresistance across histopathological sarcoma subtypes, together with their diverse tumor microenvironments, represents a confounding factor in the context of SBRT for PM from sarcoma [84]. Prospective randomized trials are ultimately needed to define the relative roles of surgery and SBRT. Until such data are available, well-designed multicenter retrospective or registry-based studies with standardized reporting—such as the ongoing SARCLUNG analysis, a multicenter propensity-matched comparison of surgery and SBRT coordinated by the German Society of Radiation Oncology—represent an important step toward clarifying their comparative effectiveness [85].

### Limitations

The most important limitations of this review are: (i) the heterogeneity of reported outcome data in the analyzed studies, limiting comprehensive statistical comparison to OS data only, and (ii) the “serious” and “critical” risk of bias in 20 of the studies included in the analysis [[22], [23], [24], [34], [38], [40], [42], [44], [49], [51], [52], [54], [58], [60], [62], [63], [64], [66], [68], [71]]. In addition, many primary studies merged and analyzed BS and STS despite their well-recognized biological and clinical differences. Consequently, our decision to analyze these populations collectively—mirroring the structure of the available evidence—introduces an inherent limitation and may obscure subtype-specific patterns of metastatic behavior and treatment response. Due to publications with higher risk of bias making up such a large proportion of the study collective, excluding them from our analyses was not feasible for fear of thinning the evidence base out too much, given the already scarce availability of reports regarding parameters such as LC, PFS, DFS and treatment-related toxicities. Thus, these infrequently reported parameters were extracted from all studies and evaluated equally, regardless of the risk of bias attached to each individual publication. The two studies judged to be at critical risk of bias were, however, excluded from the pooled analysis of altogether readily available OS data in order to improve data quality for this important endpoint. Both the heterogeneity of reporting and serious or critical risk of bias in roughly 1/3 of the available data stem from the rarity of the disease—the majority of available evidence is retrospective and derived from small patient cohorts. Moreover, because SBRT is not currently regarded as a first-line treatment for local ablation of PM from sarcoma, it is applied in a relatively small subset of (mainly unfavourable) patients, which makes direct comparison with metastasectomy challenging. This is reflected in the relatively limited number of studies (n = 17) that have investigated SBRT as a treatment strategy [[11], [12], [24], [29], [30], [31], [32], [40], [41], [48], [50], [51], [53], [55], [64], [74], [75]].

Specifically concerning the pooled analysis conducted on the OS data available to us, it must also be pointed out that our analysis assumed a constant hazard rate across studies, which simplifies comparison but may not fully capture temporal shifts in patient outcomes or imaging performance. As technological advances and evolving clinical practices can influence survival dynamics, this assumption could lead to underestimation of heterogeneity across study periods. In addition, differences in follow-up duration or censoring patterns between studies may violate the proportionality of hazards and introduce further bias.

Furthermore, substantial variability was observed in the treatment strategies employed for pulmonary metastases from sarcoma within both the surgery and SBRT subgroups. This was particularly evident for *peri*-interventional systemic therapies, which were often underreported despite being a key component in the management of metastatic sarcoma and an important confounding factor that could not be adequately addressed within the scope of this review. The heterogeneity of sarcoma histotypes and different prognoses combined with the rarity of the disease adds an additional layer of difficulty to any subgroup analyses in this context. This extends to differences in chemosensitivity across sarcoma subtypes. While systemic therapies for STS often show limited efficacy and are frequently associated with considerable toxicities – particularly in elderly patients – BS such as osteosarcomas and Ewing sarcomas have more promising systemic treatment options in the metastatic setting. Consequently, the inclusion of PM originating from both BS and STS may have introduced heterogeneity that could have influenced our findings [86].

## Conclusion

Available evidence suggests that SBRT and surgery achieve comparable oncologic outcomes for sarcoma-derived PM and that SBRT should not be viewed merely as a fallback option for patients with high surgical morbidity or contraindications to surgery. Patients at high surgical risk should preferentially receive SBRT when feasible, and current evidence further indicates that SBRT may serve as an equally valid local treatment option beyond this group. Reported toxicities appear more favorable with SBRT, though these comparisons are limited by retrospective designs, different indications in the choice of technique and incomplete reporting. Treatment choice should be individualized within a multidisciplinary sarcoma team, balancing clinical factors, patient preferences, and the specific advantages of each modality. As in other oligometastatic settings, careful patient selection is essential, and standardized recommendations for oligometastases in sarcoma are urgently needed. Well-designed multi-institutional analyses and prospective comparative trials will be key to defining the relative safety and efficacy of both modalities. Treatment decisions made within a multidisciplinary sarcoma expert setting—incorporating both clinical factors and patient preferences—remain paramount.

## Declaration of Competing Interest

The authors declare that they have no known competing financial interests or personal relationships that could have appeared to influence the work reported in this paper.

## Data Availability

This review is registered in the international prospective register of systematic reviews (PROSPERO) under: “Systematic Review on the Precision Treatment of Pulmonary Sarcoma Metastases – Stereotactic Body Radiotherapy vs Metastasectomy” (v1.3, 15th August 2025)*,* https://www.crd.york.ac.uk/PROSPERO/view/CRD42025103791. The full datasheet, including risk-of-bias analysis and the code used for study extraction has been made available on GitHub under https://github.com/maksymfritsak/Sarcoma-Lung-SBRT-VS-Metastasectomy-Systematic-Review.
