# Supplementary figures and images for: Stereotactic Body Radiotherapy vs. Metastasectomy for Soft Tissue and Bone Sarcoma Lung Metastases – A Systematic Review analyzing Safety and Efficacy
Source: Clin Transl Radiat Oncol. 2025 Dec 20;57:101097. doi: 10.1016/j.ctro.2025.101097 (PMC12804006; doi:10.1016/j.ctro.2025.101097)

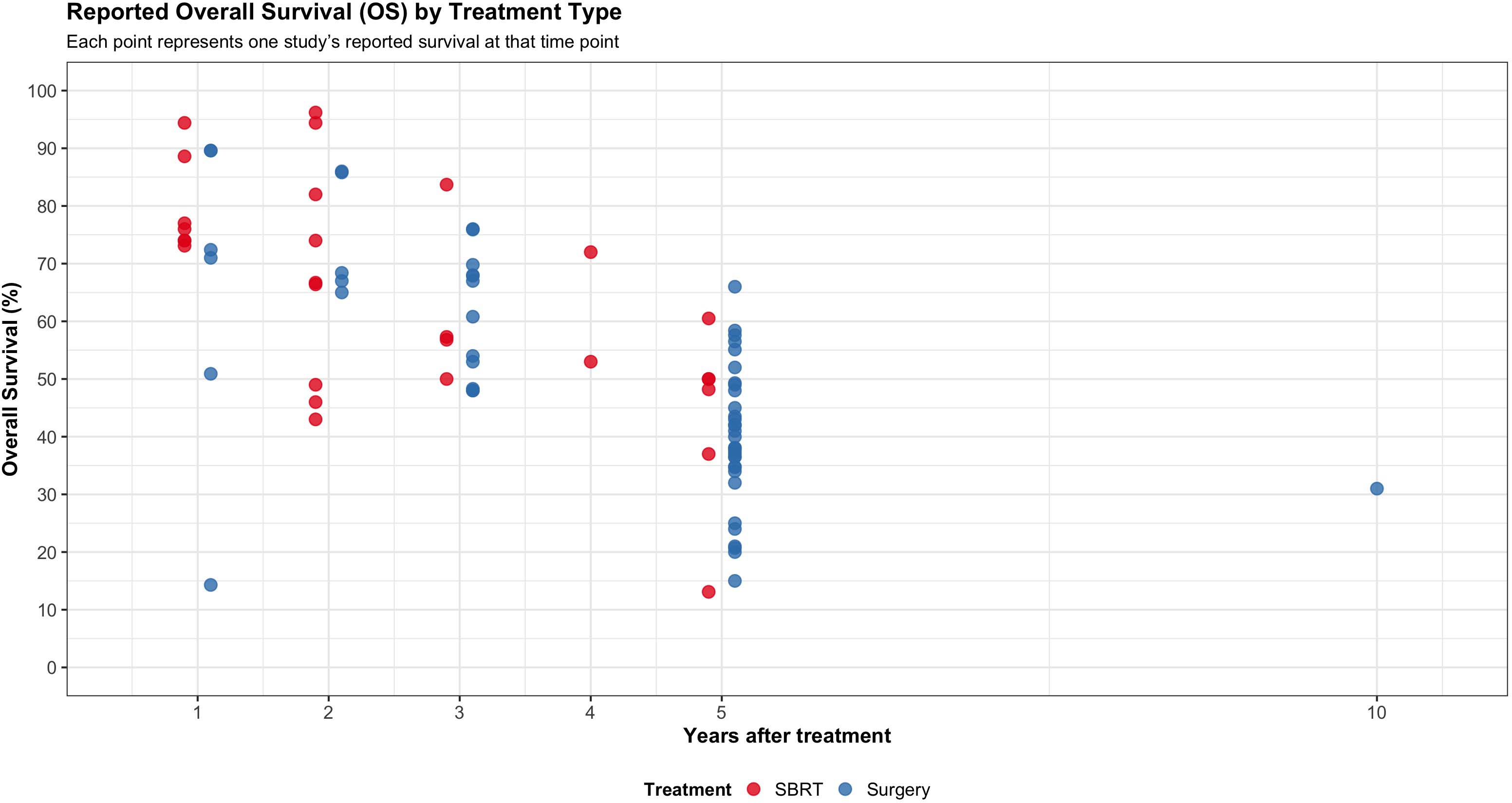

Supplement: Supplementary Fig. 1 [file mmc1.jpg]

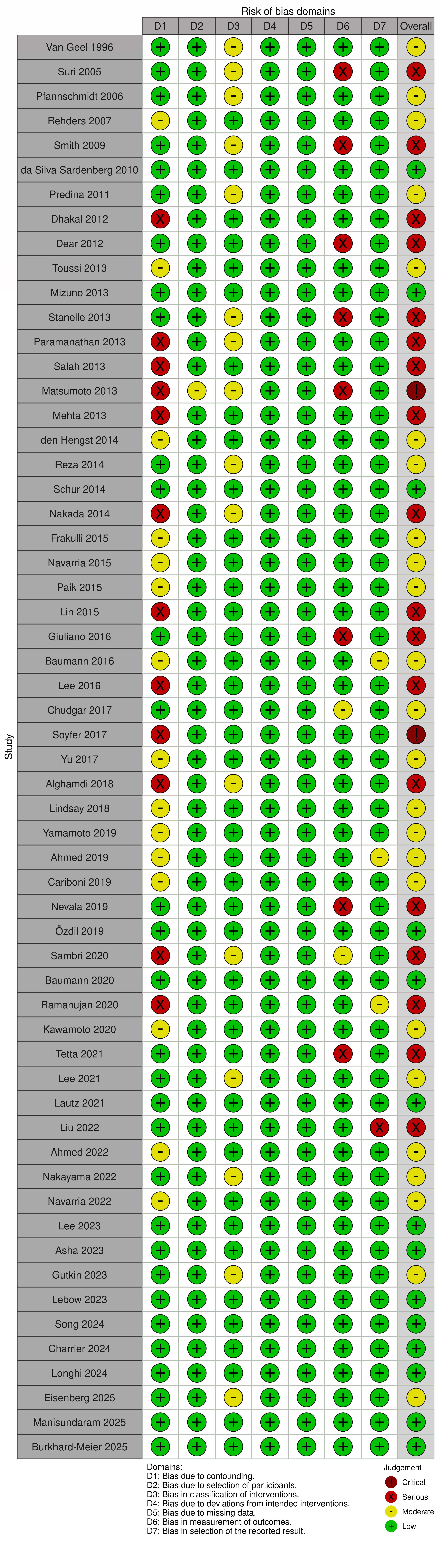

Supplement: Supplementary Fig. 2 [file mmc2.jpg]

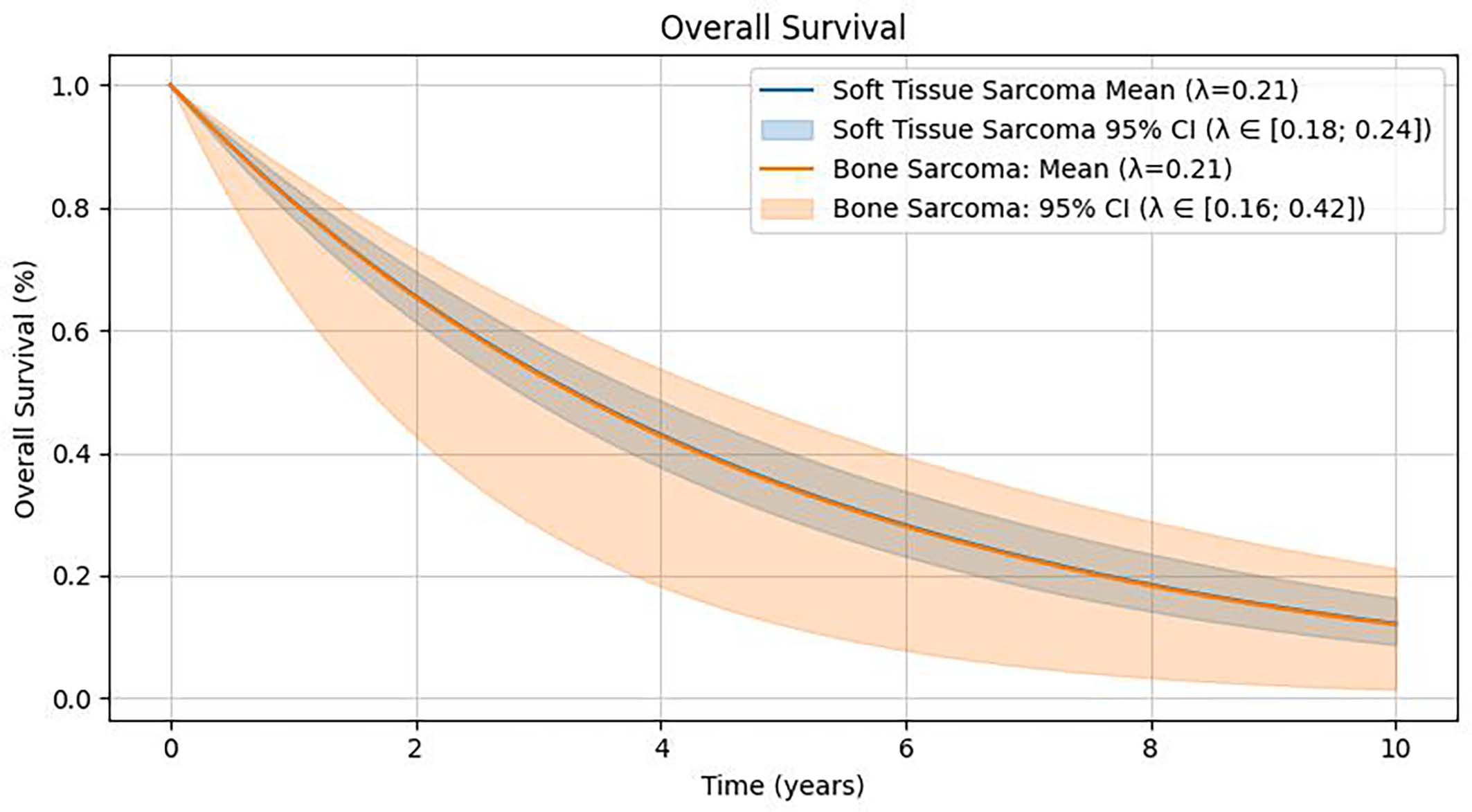

Supplement: Supplementary Fig. 3 [file mmc3.jpg]
