# Supplementary material for: Stereotactic Body Radiotherapy vs. Metastasectomy for Soft Tissue and Bone Sarcoma Lung Metastases – A Systematic Review analyzing Safety and Efficacy
Source: Clin Transl Radiat Oncol. 2025 Dec 20;57:101097. doi: 10.1016/j.ctro.2025.101097 (PMC12804006; doi:10.1016/j.ctro.2025.101097)
Supplement: Supplementary Data 1 [file mmc4.pdf]

Systematic Review on the Precision Treatment of Pulmonary Sarcoma Metastases - Stereotactic Body Radiotherapy vs Metastasectomy

Lena Kretzschmar[1], Philip Heesen[1,2], Maksym Fritsak[1,2], Sebastian Christ[1], Siyer Roohani[3,4,5,6]

[1]Department of Radiation Oncology, University Hospital Zurich and University of Zurich, Zurich, Switzerland
[2]Faculty of Medicine, University of Zurich, Zurich, Switzerland
[3]Department of Radiation Oncology, Charité-Universitätsmedizin Berlin, Corporate Member of Freie Universität Berlin and Humboldt-Universität zu Berlin, Berlin, Germany
[4]Radiation Medicine Program, Princess Margaret Cancer Centre, Department of Radiation Oncology, University of Toronto, University Health Network, Toronto, ON, Canada
[5]BIH Biomedical Innovation Academy, BIH Charité (Junior) Clinician Scientist Program, Berlin Institute of Health der Charité - Universitätsmedizin Berlin, Berlin, Deutschland
[6]German Cancer Consortium (DKTK), partner site Berlin, a partnership between DKFZ and Charité - Universitätsmedizin Berlin, Germany, Heidelberg, Germany

Review question

How do outcomes of stereotactic body radiotherapy for pulmonary sarcoma metastases compare to surgical metastasectomy?

Rationale for the Review

Pulmonary metastases are frequent in sarcoma patients, yet optimal local treatment strategies remain unclear. Surgical resection and stereotactic body radiotherapy (SBRT) are commonly used approaches, but current evidence is limited to small, heterogeneous studies. This systematic review aims to synthesise existing studies on the use of surgery or SBRT for treating lung metastases in sarcoma patients. We will focus on key clinical outcomes such as local control, progression-free survival, overall survival and treatment-related toxicity, taking histology, size and number of metastases, as well as type of intervention (e.g. dose and technique of SBRT, thoracotomy vs thoracoscopy) into account. With this review we hope to provide a clearer overview of the benefits and risks associated with each modality.

Searches

The bibliographic database PubMed (MEDLINE) will be searched using the following search term: *("radiotherapy"[MeSH Terms] OR (stereotactic AND radiotherapy) OR ("metastasectomy"[MeSH Terms]) AND ("lung neoplasms/secondary"[MeSH Terms]) AND ("sarcoma"[MeSH Terms])*. Other databases like EMBASE, Google Scholar, Scopus, Cochrane Central Register of Controlled Trials (CENTRAL) may be checked for studies that have not been made available through PubMed in a second step. No filters, language restrictions or publication time restrictions will be applied. Additionally, references of included studies will be hand searched.

Types of study to be included

We will include observational cohort and case-control studies, descriptive studies, case-reports and randomized controlled trials. Only full-texts will be considered.

Condition or domain being studied

Pulmonary metastases of sarcomas being treated with SBRT, surgical metastasectomy, or both.

Participants/population
Patients with one or more pulmonary metastasis/-es of histologically proven sarcoma treated with SBRT, surgical metastasectomy or both. Sarcoma entities will be stratified according to the 2020 WHO classification for soft tissue tumours.

Intervention(s), exposure(s)
SBRT for pulmonary sarcoma metastases.

Comparator(s)/Control
Directed metastasectomy for pulmonary sarcoma metastases.

Main outcome(s)
Local control, progression-free survival, overall survival and treatment-related toxicity.

Additional outcome(s)
Disease-free survival, disease progression outside the lung.

Data extraction (selection and coding)
Two reviewers will independently screen the titles and abstracts of studies retrieved using Rayyan to identify those that meet the inclusion criteria. Any disagreements regarding study eligibility will be resolved through discussion or, if needed, by involving a third reviewer. Data extraction will be done by two reviewers independently. Any discrepancies will be resolved through discussion or, if needed, by involving a third reviewer.

Risk of bias (quality) assessment
The bias assessment will be based on the ROBINS-I-tool (for observational studies) or the RoB-II-tool (for randomized controlled trials).

Strategy for data synthesis
Data will be qualitatively described.

Analysis of subgroups or subsets
If sufficient data are available, subgroup analyses will be conducted for different therapeutic regimens including, but not limited to, technique of SBRT, technique of surgery (e.g. thoracotomy vs. thoracoscopy), histology of primary tumour, number of metastases (e.g. oligometastatic vs. polymetastatic setting).
