# Supplementary material for: Stereotactic Body Radiotherapy vs. Metastasectomy for Soft Tissue and Bone Sarcoma Lung Metastases – A Systematic Review analyzing Safety and Efficacy
Source: Clin Transl Radiat Oncol. 2025 Dec 20;57:101097. doi: 10.1016/j.ctro.2025.101097 (PMC12804006; doi:10.1016/j.ctro.2025.101097)
Supplement: Supplementary Table 1 [file mmc5.docx]

| **Table 1:** The table summarizes study and tumor characteristics, number of metastases, treatment details, oncological outcomes and toxicities on 53 of 58 publications with n ≥ 20 patients (excluding [21 - 25]). | | | | | | | | | | | | | | | | | | | | | | | |
| --- | --- | --- | --- | --- | --- | --- | --- | --- | --- | --- | --- | --- | --- | --- | --- | --- | --- | --- | --- | --- | --- | --- | --- |
| Year of Publication / First Author | Study design | Total no. of patients | Histology | No. surg. patients / No. met. surg. | No. SBRT patients / No. met. SBRT | Surg. met. Size | SBRT met. size | Type of surg. | Extent of surg. | SBRT prescription [BED_4_ dose range] | Median FU in months | LC surg.[%] | LC SBRT [%] | PFS surg. [%] | PFS SBRT [%] | DFS surg. [%] | DFS SBRT [%] | OS surg [%]. | OS SBRT [%] | Highest surg. tox Grade | Highest SBRT tox Grade | No. surg. patients CTCAE Tox ≥ Grade 3 | No. SBRT patients CTCAE Tox ≥ Grade 3 |
| **2025 /**  **Eisenberg M** [26] | retrospective | 142 | Osteosarcoma | 142 / 689 |  | 0.5 - 1.7 cm |  | thoracoscopy; thoracotomy | lobectomy; other |  | N/A | N/A |  | N/A |  | N/A |  | N/A |  | 4 (Clavien Dindo) |  | N/A |  |
| **2025 / Manisundaram N** [27] | retrospective | 122 | Osteosarcoma, Ewing Sarcoma, Other | 122 / N/A |  | 0.7 - 2.2 cm |  | thoracoscopy; thoracotomy; sternotomy | wedge |  | 16 | N/A |  | N/A |  | N/A |  | N/A |  | 4 (CTCAE)  4 (Clavien Dindo) |  | 14 (12%) |  |
| **2025 /**  **Burkhard-Meier A** [28] | retrospective | 77 | Leiomyosarcoma, Synovial Sarcoma, UPS | 77 / N/A |  | 0.3 - 11.2 cm |  | thoracoscopy; thoracotomy | N/A |  | 43 | N/A |  | N/A |  | 19.0 (4 yr) |  | 85.8 (2 yr) / 49.3  (5 yr) |  | 3 (CTCAE)  3 (Clavien Dindo) |  | 2 (3%) |  |
| **2024 /**  **Charrier T** [29] | retrospective | 192 | Osteosarcoma, Chondrosarcoma, Synovial Sarcoma, Leiomyosarcoma, Fibrosarcoma, Other | 192 / N/A |  | N/A |  | thoracoscopy; thoracotomy | wedge; segmentectomy; lobectomy |  | 32 | N/A |  | N/A |  | N/A |  | 89.6 (1 yr) / 69.8 (3 yr) / 57.6 (5 yr) |  | N/A |  | N/A |  |
| **2024 / Longhi A** [11] | retrospective | 102 | Osteosarcoma, Ewing Sarcoma, Chondrosarcoma, UPS, Chondrosarcoma, Giant Cell Tumor, Leiomyosarcoma, Synovial Sarcoma, Liposarcoma, MPNST, Myxofibrosarcoma, Triton Tumor, ASPS, Malignant Myoepithelioma, Solitary Malignant Fibrous Tumour |  | 102 / 276 |  | 0.7 - 5 cm |  |  | 3 – 8 fx,  30 - 60 Gy total (IDL 80%) [BED_4_ 105 - 142.5 Gy] | 58 |  | 86.0 (1 yr) / 78.0  (2 yrs) |  | N/A |  | N/A |  | 37.0 (5 yr) |  | 2 (CTCAE)  3 (Clavien Dindo) |  | 0 |
| **2024 / Song Y** [30] | retrospective | 97 | UPS, Leiomyosarcoma, Dedifferentiated Liposarcoma, Osteosarcoma, Other | 61 / N/A | 36 / 54 | N/A | 0.1 - 75.8 cc | N/A | wedge; segmentectomy; lobectomy | 3 - 10 fx, 48 - 60 Gy total (IDL N/A)  [BED_4_ 113 - 240 Gy] | 19 (LC) / 45 (OS) | 89.2 (3 yr) | 92.3 (3 yr) | N/A | N/A | N/A | N/A | 75.9 (3 yr) | 57.3 (3 yr) | N/A | 2 (CTCAE)  1 (Clavien Dindo) | N/A | 0 |
| **2023 / Lebow ES** [31] | retrospective | 66 | Leiomyosarcoma, UPS, Liposarcoma, Osteosarcoma, Synovial Sarcoma, Other |  | 66 / 95 |  | 0.2 - 375.8 cc |  |  | 1 - 8 fx,  24 - 60 Gy total (IDL N/A)  [BED_4_ 75 - 297 Gy] | 36 |  | 96.9 (1 yr) / 92.6  (2 yr) |  | N/A |  | N/A |  | 74.0 (1 yr) / 49.0  (2 yr) |  | 2 (CTCAE)  1 (Clavien Dindo) |  | 0 |
| **2023 / Asha W** [32] | retrospective | 50 | Angiosarcoma, Chondrosarcoma, Leiomyosarcoma, Liposarcoma, MPNST, Myxofibrosarcoma, Osteosarcoma, Synovial Sarcoma, UPS, Other |  | 50 / 109 |  | N/A |  |  | 1 - 5 fx,  30 - 60 Gy total (IDL N/A) [BED_4_ 192 - 323 Gy] | 20 |  | 96.0 (1 yr) / 88.0  (3 yr) |  | N/A |  | N/A |  | 77.0 (1 yr) / 50.0  (3 yr) |  | 3 (CTCAE)  1 (Clavien Dindo) |  | 1 (2%) |
| **2023 / Lee TH** [33] | retrospective | 39 | Leiomyosarcoma, Osteosarcoma, UPS, Liposarcoma, Synovial Sarcoma, Chondrosarcoma, Myxofibrosarcoma, Spindle Cell Sarcoma, ASPS, Ewing Sarcoma, Malignant Glomus Tumor, MPNST |  | 39 / 71 |  | 0.3 - 3.6 cm |  |  | 1 - 5 fx,  20 - 60 Gy total (IDL N/A)  [BED_4_ 70 - 360 Gy] | 27 |  | 100.0  (1 yr) / 84.6 (2 yr) / 78.1  (3 yr) |  | 52.7 (1 yr) / 20.6  (2 yr) / 12.9 (3 yr) |  | N/A |  | 94.4 (1 yr) / 94.4  (2 yr) / 83.7 (3 yr) |  | 3 (CTCAE)  2 (Clavien Dindo) |  | 1 (3%) |
| **2022 / Ahmed G** [34] | retrospective | 125 | Osteosarcoma | 125 / 561 |  | N/A |  | thoracotomy | wedge; lobectomy |  | N/A | N/A |  | N/A |  | 18.1 (5 yr) |  | 36.5 (5 yr) |  | N/A |  | N/A |  |
| **2022 / Liu Z** [35] | retrospective | 125 | Osteosarcoma | 59 / 112 |  | up to 5.2 cm |  | thoracoscopy; thoracotomy; other | wedge; lobectomy; other |  | 72 | N/A |  | N/A |  | N/A |  | 68.4 (2 yr) |  | N/A |  | N/A |  |
| **2022 / Nakayama S** [36] | retrospective | 66 | Synovial Sarcoma, UPS, Liposarcoma, Myxofibrosarcoma, Leiomyosarcoma, Dermatofibrosarcoma, Ewing Sarcoma, MPNST, Epithelioid Sarcoma, ASPS, Angiosarcoma | 66 / N/A |  | 0.1 - 6 cm |  | thoracoscopy; thoracotomy | wedge; segmentectomy; lobectomy |  | 28 | N/A |  | N/A |  | N/A |  | 45.0 (5 yr) |  | 2 (CTCAE)  2 (Clavien Dindo) |  | 0 |  |
| **2022 / Navarria P** [12] | prospective interventional (non-randomized) | 44 | Leiomyosarcoma, Spindle Cell Sarcoma, Synovial Sarcoma, Solitary fibrous, Chondrosarcoma, Myxofibrosarcoma, Ewing Sarcoma, UPS, Liposarcoma, Rhabdomyosarcoma, ASPS |  | 44 / 71 |  | 0.36 - 62 cc |  |  | 1 - 8 fx,  30 - 60 Gy total (IDL N/A)  [BED_4_ 172.5 - 360 Gy] | 48 |  | 98.5 (1 yr) / 98.5  (2 yr) / 98.5 (3 yr) / 93.1  (4 yr) / 93.1 (5 yr) |  | N/A |  | 50.0 (1 yr) / 19.5  (2 yr) / 11.7 (3 yr) / 11.7  (4 yr) / 11.7 (5 yr) |  | 88.6 (1 yr) / 66.7  (2 yr) / 56.8 (3 yr) / 53.0  (4 yr) / 48.2 (5 yr) |  | 2 (CTCAE)  1 (Clavien Dindo) |  | 0 |
| **2021 / Lee RM** [37] | retrospective | 352 | UPS, Leiomyosarcoma, Synovial Sarcoma, Other | 352 / N/A |  | N/A |  | thoracoscopy; thoracotomy | wedge; lobectomy |  | 26 | N/A |  | N/A |  | N/A |  | 42.0 (5 yr) |  | N/A |  | N/A |  |
| **2021 / Lautz TB** [38] | retrospective | 202 | Osteosarcoma | 202 / N/A |  | N/A |  | thoracoscopy; thoracotomy | N/A |  | 45 | N/A |  | N/A |  | 25.0 (5 yr) |  | 49.0 (5 yr) |  | 5 (CTCAE)  5 (Clavien Dindo) |  | N/A |  |
| **2021 / Tetta C** [39] | retrospective | 164 | Synovial Sarcoma, UPS, Myxofibrosarcoma, Extraskeletal Myxoid Sarcoma Chondrosarcoma, Epithelioid , Leiomyosarcoma, Extra-skeletal Ewing Sarcoma, MPNST, Clear Cell Sarcoma, Undifferentiated Sarcoma, Other | 164 / 447 |  | N/A |  | thoracoscopy; thoracotomy | wedge; other |  | 61 | N/A |  | N/A |  | N/A |  | N/A |  | N/A |  | N/A |  |
| **2020 / Kawamoto T** [40] | retrospective | 98 | Leiomyosarcoma, UPS, Synovial Sarcoma, Myxofibrosarcoma, Liposarcoma, MPNST, Rhabdomyosarcoma, Clear Cell Sarcoma, Other | 41 / N/A |  | N/A |  | thoracoscopy; thoracotomy | wedge; segmentectomy; lobectomy |  | N/A | N/A |  | N/A |  | N/A |  | 89.6 (1 yr) / 67.9  (3 yr) / 56.5 (5 yr) |  | N/A |  | N/A |  |
| **2020 / Sambri A** [41] | retrospective | 61 | Chondrosarcoma | 29 / N/A | 32 / N/A | N/A | N/A | N/A | N/A | N/A | 83 | N/A | N/A | N/A | N/A | N/A | N/A | 55.1 (5 yr) | 13.1 (5 yr) | N/A | N/A | N/A | N/A |
| **2020 / Baumann BC** [42] | retrospective | 44 | Leiomyosarcoma, UPS, Chondrosarcoma, Liposarcoma, Synovial Sarcoma cell, Fibro, Solitary fibrous tumour, Epithelioid cell, Carcino, Desmoplastic small round cell tumour, Ewing Sarcoma, ASPS, Osteosarcoma, MPNST |  | 44 / 56 |  | 0.2 - 235 cc |  |  | 3 - 5 fx,  24 - 50 Gy total (IDL N/A)  [BED_4_ 60 - 206.3 Gy] | 16 |  | 96.0 (1 yr) / 90.0  (2 yr) |  | N/A |  | N/A |  | 74.0 (1 yr) / 46.0  (2 yr) |  | 2 (CTCAE)  1 (Clavien Dindo) |  | 0 |
| **2020 / Ramanujan V** [43] | retrospective | 37 | Osteosarcoma | 37 / N/A |  | N/A |  | thoracoscopy; thoracotomy | N/A |  | N/A | N/A |  | N/A |  | N/A |  | 86.0 (2 yr) / 60.8  (3 yr) / 20.7 (5 yr) |  | N/A |  | N/A |  |
| **2019 / Cariboni U** [44] | retrospective | 154 | Synovial Sarcoma, Chondrosarcoma, Leiomyosarcoma, Liposarcoma, UPS, MPNST, Myxofibrosarcoma, Ewing Sarcoma, Other | 154 / N/A |  | N/A |  | thoracoscopy; thoracotomy; other | wedge; segmentectomy; lobectomy; pneumonectomy |  | 24 | N/A |  | N/A |  | 17.2 (5 yr) |  | 36.5 (5 yr) |  | N/A |  | N/A |  |
| **2019 / Nevala R** [45] | retrospective | 130 | UPS, Liposarcoma, Synovial Sarcoma, Leiomyosarcoma, MPNST, Fibrosarcoma, Neurofibrosarcoma, Myxofibrosarcoma, Epithelioid Sarcoma, Other | 74 / N/A |  | N/A |  | thoracoscopy; thoracotomy | N/A |  | 29 | N/A |  | N/A |  | N/A |  | 20.0 (5 yr) |  | 5 (CTCAE)  5 (Clavien Dindo) |  | N/A |  |
| **2019 / Ahmed G** [46] | retrospective | 88 | Osteosarcoma | 88 / N/A |  | N/A |  | thoracotomy | wedge; lobectomy |  | 35 | N/A |  | 25.0 (5 yr) |  | N/A |  | 38.1 (5 yr) |  | N/A |  | N/A |  |
| **2019 / Özdil A** [47] | retrospective | 69 | Osteosarcoma, Ewing Sarcoma, Synovial Sarcoma, Other | 69 / N/A |  | 0.2 - 14 cm |  | thoracoscopy; thoracotomy | wedge; segmentectomy; lobectomy |  | 35 | N/A |  | N/A |  | 38.0 (5 yr) |  | 48.0 (5 yr) |  | N/A |  | N/A |  |
| **2019 / Yamamoto Y** [48] | retrospective | 44 | Osteosarcoma, Synovial Sarcoma , UPS, Ewing Sarcoma, Myxofibrosarcoma, Rhabdomyosarcoma, MPNST, Other | 44 / N/A |  | 0.4 - 11.5 cm |  | thoracoscopy; thoracotomy | wedge; segmentectomy; lobectomy |  | 130 | N/A |  | N/A |  | N/A |  | 43.5 (5 yr) |  | N/A |  | N/A |  |
| **2018/ Lindsay AD** [49] | retrospective | 44 | UPS, Synovial Sarcoma, Leiomyosarcoma, Ewing Sarcoma, Hemangiopericytoma, Myxofibrosarcoma, Spindle Cell Sarcoma, Chondrosarcoma, Liposarcoma, Osteosarcoma |  | 44 / 117 |  | 0.8 - 7 cm |  |  | 5 - 12 fx, 30 - 55 Gy total (IDL N/A)  [BED_4_ 67.5 - 175 Gy] | 14 |  | 95.0 (1 yr) |  | N/A |  | N/A |  | 82.0 (2 yr) / 50.0  (5 yr) |  | 3 (CTCAE)  3 (Clavien Dindo) |  | 1 (2%) |
| **2018 / Alghamdi AA** [50] | retrospective | 38 | Ewing Sarcoma, Fibrosarcoma, Leiomyosarcoma, Osteosarcoma, Synovial Sarcoma, Other | 22 / N/A |  | N/A |  | thoracoscopy; thoracotomy | N/A |  | 26 | N/A |  | N/A |  | N/A |  | 41.0 (5 yr) |  | N/A |  | N/A |  |
| **2017 / Chudgar NP** [51] | registry-based | 539 | Leiomyosarcoma, UPS, Synovial Sarcoma, Fibrosarcoma, Liposarcoma, MPNST, Other | 539 / N/A |  | N/A |  | thoracoscopy; thoracotomy; sternotomy | wedge; lobectomy; pneumonectomy |  | 27 | N/A |  | N/A |  | N/A |  | 34.0 (5 yr) |  | N/A |  | N/A |  |
| **2017 / Yu W** [52] | retrospective | 73 | Osteosarcoma | 40 / N/A | 33 / N/A | 0.4 - 2.7 cm | 0.4 - 2.7 cm | thoracotomy | wedge; lobectomy | 10 fx,  50 Gy total (IDL 50%)  [BED_4_ 113 Gy] | 36 | N/A | N/A | 27.5 (4 yr) | 21.2 (4 yr) | N/A | N/A | N/A | N/A | 4 (CTCAE)  4 (Clavien Dindo) | 2 (CTCAE)  1 (Clavien Dindo) | N/A | 0 |
| **2017 / Soyfer V** [53] | retrospective | 22 | Spindle Cell Sarcoma, Fibro, UPS, Synovial Sarcoma, Leiomyosarcoma, Osteosarcoma, Other |  | 22 / 53 |  | N/A |  |  | 3 - 4 fx,  24 - 60 Gy total (IDL N/A)  [BED_4_ 72 - 360 Gy] | 94 |  | 96.0 (8 yr) |  | N/A |  | N/A |  | 50.0 (5 yr) |  | 3 (CTCAE) |  | 1 (5%) |
| **2016 / Giuliano K** [54] | retrospective | 53 | Leiomyosarcoma, UPS, Synovial Sarcoma, Spindle Cell Sarcoma, Rhabdomyosarcoma, Fibrosarcoma, MPNST, Epithelioid, Liposarcoma, Neurogenic, Endometrial Stromal Sarcoma, Other | 53 / 92 |  | up to 12 cm |  | thoracoscopy; thoracotomy; sternotomy; other | wedge; segmentectomy; lobectomy; other |  | 85 | N/A |  | N/A |  | N/A |  | 68.0 (3 yr) |  | 5 (CTCAE)  5 (Clavien Dindo) |  | N/A |  |
| **2016 / Baumann BC** [55] | retrospective | 30 | Leiomyosarcoma, UPS, Chondrosarcoma, Liposarcoma, Synovial Sarcoma, Fibrosarcoma, Malignant Fibrous Sarcoma, Epithelioid Cell Sarcoma, Carcinosarcoma, Desmoplastic Small Round Cell Sarcoma, Ewing Sarcoma, ASPS |  | 30 / 39 |  | 0.5 - 8.1 cm |  |  | 4 - 5 fx,  24 - 50 Gy total (IDL N/A)  [BED_4_ 60 - 206.3 Gy] | 16 |  | 94.0 (1 yr) / 86.0 (2 yr) |  | N/A |  | N/A |  | 76.0 (1 yr) / 43.0  (2 yr) |  | 2 (CTCAE)  1 (Clavien Dindo) |  | 0 |
| **2016 / Lee K** [56] | retrospective | 29 | Synovial Sarcoma | 29 / N/A |  | N/A |  | thoracoscopy; sternotomy | segmentectomy; lobectomy; pneumonectomy |  | 68 | N/A |  | N/A |  | N/A |  | 58.4 (5 yr) |  | 5 (CTCAE)  5 (Clavien Dindo) |  | N/A |  |
| **2015 / Lin AY** [57] | registry-based | 155 | Leiomyosarcoma, Osteosarcoma, Synovial Sarcoma, Chondrosarcoma, Liposarcoma, UPS, Ewing Sarcoma, MPNST, ASPS, Rhabdomyosarcoma, Other | 155 / N/A |  | 0.3 - 16 cm |  | thoracoscopy; thoracotomy; sternotomy; other | wedge; segmentectomy; lobectomy; pneumonectomy |  | 39 | N/A |  | N/A |  | 7.7 (5 yr) |  | 34.8 (5 yr) |  | N/A |  | N/A |  |
| **2015 / Navarria P** [58] | prospective observational | 28 | Leiomyosarcoma, Synovial Sarcoma, Spindle Cell Sarcoma, Other |  | 28 / 51 |  | 3.2 - 187.2 cc |  |  | 1 - 8 fx,  30 - 60 Gy total (IDL N/A)  [BED_4_ 172.5 - 360 Gy] | 14 |  | 96.2 (2 yr) |  | N/A |  | N/A |  | 96.2 (2 yr) / 60.5  (5 yr) |  | 2 (CTCAE)  1 (Clavien Dindo) |  | 0 |
| **2015 / Frakulli R** [59] | retrospective | 24 | Spindle Cell Sarcoma, Ewing Sarcoma, Chondrosarcoma, Osteosarcoma, Liposarcoma, Leiomyosarcoma, Giant Cell Tumor, Schwannoma, Synovial Sarcoma |  | 24 / 68 |  | 0.51 - 295.7 cc |  |  | 3 - 8 fx, 30 - 60 Gy total (IDL 80%)  [BED_4_ 105 - 172.5 Gy] | 49 |  | 88.2 (1 yr) / 85.9  (2 yr) |  | N/A |  | N/A |  | 73.1 (1 yr) / 66.4  (2 yr) |  | 2 (CTCAE)  1 (Clavien Dindo) |  | 0 |
| **2014 / Reza J** [60] | retrospective | 118 | UPS, Fibro, Osteosarcoma, Ewing Sarcoma, Synovial Sarcoma, Spindle Cell Sarcoma, Leiomyosarcoma, MPNST, Chondrosarcoma, Rhabdomyosarcoma, Liposarcoma, Giant Cell Tumor, Other | 118 / N/A |  | N/A |  | thoracoscopy; thoracotomy; other | wedge; segmentectomy; lobectomy; pneumonectomy |  | N/A | N/A |  | N/A |  | 17.0 (5 yr) |  | 48.0 (3 yr) / 42.0  (5 yr) / 31.0 (10 yr) |  | 5 (CTCAE)  5 (Clavien Dindo) |  | N/A |  |
| **2014 / den Hengst WA** [61] | prospective interventional (non-randomized) | 50 | Osteosarcoma, Chondrosarcoma, Fibrosarcoma, Synovial Sarcoma, Leiomyosarcoma, Other | 20 / N/A |  | N/A |  | thoracotomy | other |  | 24 | 90.0 (3 yr) |  | N/A |  | 27.0 (3 yr) |  | 48.0 (3 yr) |  | 4 (CTCAE)  4 (Clavien Dindo) |  | N/A |  |
| **2014 / Schur S** [62] | retrospective | 46 | Leiomyosarcoma, Myxofibrosarcoma, Synovial Sarcoma, UPS, Liposarcoma, Malignant mesenchymoma, Myxofibrosarcoma, Malignant solitary fibrous tumour, Rhabdomyosarcoma, MPNST, Endometrial Stromal Sarcoma, Extraskeletal Myxoid Sarcoma Chondrosarcoma, Angiosarcoma,  Myofibroblastic Sarcoma, Undifferentiated Epithelioid Sarcoma | 46 / 322 |  | N/A |  | thoracoscopy; thoracotomy | N/A |  | 32 | N/A |  | N/A |  | N/A |  | 32.0 (5 yr) |  | 3 (CTCAE)  3 (Clavien Dindo) |  | N/A |  |
| **2013 / Salah S** [63] | retrospective | 73 | Osteosarcoma, Other | 30 / 195 |  | N/A |  | thoracotomy | segmentectomy; lobectomy; pneumonectomy |  | 25 | N/A |  | N/A |  | N/A |  | N/A |  | 5 (CTCAE)  5 (Clavien Dindo) |  | 4 (13%) |  |
| **2013 / Mizuno T** [64] | retrospective | 52 | Osteosarcoma, UPS, Liposarcoma, Synovial Sarcoma, Other | 52 / N/A |  | N/A |  | thoracoscopy; thoracotomy | wedge; segmentectomy; lobectomy; other |  | 33 | N/A |  | N/A |  | N/A |  | 50.9 (5 yr) |  | N/A |  | N/A |  |
| **2013 / Stanelle EJ** [65] | retrospective | 41 | Synovial Sarcoma | 31 / N/A |  | 0.4 - 19 cm |  | thoracoscopy; thoracotomy; sternotomy | wedge; segmentectomy; lobectomy; pneumonectomy |  | N/A | N/A |  | N/A |  | N/A |  | 65.0 (2 yr) / 24.0  (5 yr) |  | N/A |  | N/A |  |
| **2013 / Toussi MS** [66] | retrospective | 34 | UPS, Fibrosarcoma, Synovial Sarcoma, Liposarcoma, MPNST, Dermatofibrosarcoma, Leiomyosarcoma | 34 / N/A |  | 1 - 10 cm |  | thoracotomy | wedge; segmentectomy; lobectomy; pneumonectomy |  | 26 | N/A |  | N/A |  | 56.7 (2 yr) |  | 67.0 (2 yr) |  | N/A |  | N/A |  |
| **2013 / Matsumoto I** [67] | retrospective | 29 | Osteosarcoma | 29 / N/A |  | 0.8 - 4.2 cm |  | thoracotomy; sternotomy | N/A |  | 26 | N/A |  | N/A |  | N/A |  | 72.4 (1 yr) / 48.3  (3 yr) / 37.1 (5 yr) |  | N/A |  | N/A |  |
| **2012 / Dear RF** [68] | retrospective | 114 | Osteosarcoma, Chondrosarcoma, Ewing Sarcoma, UPS, Leiomyosarcoma, Synovial Sarcoma, Other | 114 / N/A |  | N/A |  | thoracoscopy; thoracotomy; other | wedge; segmentectomy; lobectomy; pneumonectomy; other |  | N/A | N/A |  | N/A |  | 36.0 (1 yr) |  | 71.0 (1 yr) / 53.0  (3 yr) / 43.0 (5 yr) |  | N/A |  | N/A |  |
| **2012 / Dhakal S** [69] | retrospective | 52 | Leiomyosarcoma, UPS, Synovial Sarcoma, Liposarcoma, Other | 16 / N/A | 14 / 74 | N/A | N/A | N/A | N/A | 10 fx, 50 Gy total (IDL 80%)  [BED_4_ 113 Gy] | 11 | N/A | 88.0 (2 yr) / 82.0  (3 yr) | N/A | N/A | N/A | N/A | N/A | N/A | N/A | 2 (CTCAE) | N/A | 0 |
| **2011 / Predina JD** [5] | retrospective | 48 | UPS, Leiomyosarcoma, Synovial Sarcoma, Liposarcoma, Ewing Sarcoma, GIST, Dermatofibrosarcoma, Neurofibrosarcoma, Hemangiosarcoma, Epithelioid Sarcoma, Clear Cell Sarcoma, Endometrial Sarcoma | 48 / N/A |  | N/A |  | thoracoscopy; thoracotomy; sternotomy | N/A |  | N/A | N/A |  | N/A |  | 17.0 (3 yr) / 10.0  (5 yr) |  | 67.0 (3 yr) / 52.0  (5 yr) |  | N/A |  | N/A |  |
| **2010 / da Silva Sardenberg RA** [70] | retrospective | 77 | UPS, Liposarcoma, Fibrosarcoma, Synovial Sarcoma, Leiomyosarcoma, Spindle Cell Sarcoma, Epithelioid Sarcoma, Rhabdomyosarcoma, Angiosarcoma, Hemangiopericytoma, Other | 77 / 268 |  | N/A |  | thoracotomy | wedge; segmentectomy; lobectomy |  | 37 | N/A |  | N/A |  | N/A |  | 34.7 (5 yr) |  | N/A |  | N/A |  |
| **2009 / Smith R** [71] | retrospective | 94 | UPS, Synovial Sarcoma, Leiomyosarcoma, Liposarcoma, Other | 94 / N/A |  | N/A |  | N/A | wedge; lobectomy; pneumonectomy |  | 60 | N/A |  | N/A |  | 5.0 (5 yr) |  | 15.0 (5 yr) |  | 5 (CTCAE)  5 (Clavien Dindo) |  | N/A |  |
| **2007 / Rehders A** [72] | registry-based | 61 | UPS, Leiomyosarcoma, MPNST, Liposarcoma, Synovial Sarcoma, ASPS , Fibrosarcoma, Other | 61 / N/A |  | N/A |  | thoracotomy; sternotomy | wedge; lobectomy |  | 60 | N/A |  | N/A |  | N/A |  | 25.0 (5 yr) |  | N/A |  | N/A |  |
| **2006 / Pfannschmidt J** [73] | retrospective | 50 | Rhabdomyosarcoma, UPS, Synovial Sarcoma, Fibrosarcoma, Liposarcoma, Hemangiopericytoma, Neuro-  Ectodermal Sarcoma,  Ewing Sarcoma, Chondrosarcoma, Leiomyosarcoma, Other | 50 / N/A |  | N/A |  | thoracoscopy; thoracotomy; sternotomy | wedge; segmentectomy; lobectomy; pneumonectomy |  | 34 | N/A |  | N/A |  | N/A |  | 37.6 (5 yr) |  | N/A |  | N/A |  |
| **2005 / Suri RM** [74] | retrospective | 103 | UPS | 103 / N/A |  | N/A |  | thoracoscopy; thoracotomy; sternotomy | wedge; segmentectomy; lobectomy; pneumonectomy; other |  | 18 | N/A |  | N/A |  | N/A |  | 21.0 (5 yr) |  | 5 (CTCAE)  5 (Clavien Dindo) |  | N/A |  |
| **1996 / Van Geel AN** [75] | retrospective | 254 | UPS, Synovial Sarcoma, Leiomyosarcoma, Liposarcoma, Fibrosarcoma, Other | 254 / N/A |  | N/A |  | thoracotomy; sternotomy | N/A |  | 30 | N/A |  | N/A |  | 42.0 (3 yr) / 35.0  (5 yr) |  | 54.0 (3 yr) / 38.0  (5 yr) |  | 5 (CTCAE)  5 (Clavien Dindo) |  | N/A |  |

| **Abbreviations:** ASPS = Alveolar Soft Part Sarcoma, BED_4_ = Biologically Effective Dose at alpha/beta ratio 4, CTCAE = Common Terminology Criteria for Adverse Events, DFS = Disease-free survival, FU = Follow-up, LC = Local control, met. = metastasis/-es, MPNST = Malignant peripheral nerve sheath tumor, N/A = data not available, no. = number, OS = Overall survival, PFS = Progression-free survival, SBRT = Stereotactic body radiotherapy, surg. = surgery, tox. = toxicity, UPS = undifferentiated pleomorphic sarcoma, yr. = year(s). |
| --- |
